# MicroRNAs-Based Nano-Strategies as New Therapeutic Approach in Multiple Myeloma to Overcome Disease Progression and Drug Resistance

**DOI:** 10.3390/ijms21093084

**Published:** 2020-04-27

**Authors:** Vanessa Desantis, Ilaria Saltarella, Aurelia Lamanuzzi, Assunta Melaccio, Antonio Giovanni Solimando, Maria Addolorata Mariggiò, Vito Racanelli, Angelo Paradiso, Angelo Vacca, Maria Antonia Frassanito

**Affiliations:** 1 Department of Biomedical Sciences and Human Oncology, Unit of Internal Medicine and Clinical Oncology, University of Bari “Aldo Moro”, 70124 Bari, Italy; vanessa86.desantis@gmail.com (V.D.); ilaria.saltarella@libero.it (I.S.); aurelia.lamanuzzi@libero.it (A.L.); assuntamel@hotmail.it (A.M.); antonio.solimando@uniba.it (A.G.S.); vito.racanelli1@uniba.it (V.R.); 2 Experimental Medical Oncology, IRCS Istituto Tumori "Giovanni Paolo II" of Bari, 70124 Bari, Italy; a.paradiso@oncologico.bari.it; 3 Department of Biomedical Sciences and Human Oncology, Unit of General Pathology, University of Bari “Aldo Moro”, 70124 Bari, Italy; mariaaddolorata.mariggio@uniba.it (M.A.M.); antofrassanito@gmail.com (M.A.F.)

**Keywords:** microRNAs, exosomes, lipid-based nanocarriers, polymer-based nanocarriers, multiple myeloma

## Abstract

MicroRNAs (miRNAs, or miRs) are single-strand short non-coding RNAs with a pivotal role in the regulation of physiological- or disease-associated cellular processes. They bind to target miRs modulating gene expression at post-transcriptional levels. Here, we present an overview of miRs deregulation in the pathogenesis of multiple myeloma (MM), and discuss the potential use of miRs/nanocarriers association in clinic. Since miRs can act as oncogenes or tumor suppressors, strategies based on their inhibition and/or replacement represent the new opportunities in cancer therapy. The miRs delivery systems include liposomes, polymers, and exosomes that increase their physical stability and prevent nuclease degradation. Phase I/II clinical trials support the importance of miRs as an innovative therapeutic approach in nanomedicine to prevent cancer progression and drug resistance. Results in clinical practice are promising.

## 1. Introduction

Multiple myeloma (MM) is an incurable hematologic malignancy characterized by the clonal accumulation of monotypic paraprotein-secreting cells (MM cells) in the bone marrow (BM) [1]. Its pathophysiology depends on different oncogenic events at MM cell level as well as on extracellular factors within the BM microenvironment (BMME) [2]. In the last years, the use of new drugs, i.e., proteasome inhibitors, immune-modulatory drugs and immunotherapy, improved MM response rate, thus increasing the patients’ survival. Nevertheless, MM remains an incurable disease that evolves into a drug resistant phase and results in patient death [3].

The miRs are highly conserved small non-coding single-strand RNA molecules (18–25 nucleotides length) that lack mRNA complementarity. They modulate gene expression at post-transcriptional levels by binding to the 3′ untranslated region (3′UTR) of mRNAs targets that induce their degradation, translational repression, and/or deadenylation [4,5]. These small RNA oligonucleotides are implicated in several physiological and pathological conditions, including cancer diseases. As a single miR can interact with many mRNAs, miRs simultaneously modulate numerous cellular signaling pathways resulting in cell growth, proliferation, metastasis, and drug resistance [6,7,8].

Deregulation of miRs expression has been documented in MM [9,10]. MM cells can express miRs at lower or higher levels compared to normal conditions [11,12] and these miRs act as tumor suppressors or oncogenes. Since the tumor suppressors’ miRs expression is lower in cancer, the reinstatement of their normal levels by miRs replacement strategy (“miRs mimics”) may provide therapeutic benefits. In contrast, overexpressed miRs (“oncomiRs”) are oncogenes that promote tumor growth by downregulation of tumor suppressor genes [13]. The therapeutic strategy of the miRs inhibition uses the delivery of specific miRs antagonists, also known as antagomiRs [14]

For clinical application, miRs need a delivery system (“nanocarriers”) to improve their efficacy in vivo and to increase the therapeutic index. Nanocarriers protect miRs from the nucleases degradation and prevent their molecular instability [15,16,17]. The delivery systems are specifically designed to transfer high concentration of active miRs to target cells by endocytosis. Nanotechnology has progressed because of new non-viral delivery systems, i.e., lipoplexes, stable nucleic acid lipid particles (SNALPs), cationic lipids, cationic polymers, and exosomes. The combination between conventional chemotherapeutic drugs and miRs has improved the therapeutic outcome in terms of synergic effects in the inhibition of tumor growth, reversion of chemoresistance, suppression of angiogenesis, apoptosis, and induction of immune response [18,19,20].

Here, we focus on miRs deregulation in MM and on their role as an innovative nano-strategy to hinder disease progression and drug resistance.

## 2. miRs Biogenesis and Mechanism of Action

The miRs are encoded in introns of coding/non-coding transcripts and only few miRs loci are located within exons of coding transcripts [5]. Several miRs loci are near to each other and constitute a single polycistronic transcription unit that encodes mature miRs clusters with similar expression profiles and biological functions [21,22]. The miRs may share the promoter of the host gene or may have their own promoter with upstream regulatory elements that modulates their expression [5,23].

**Figure 1:**
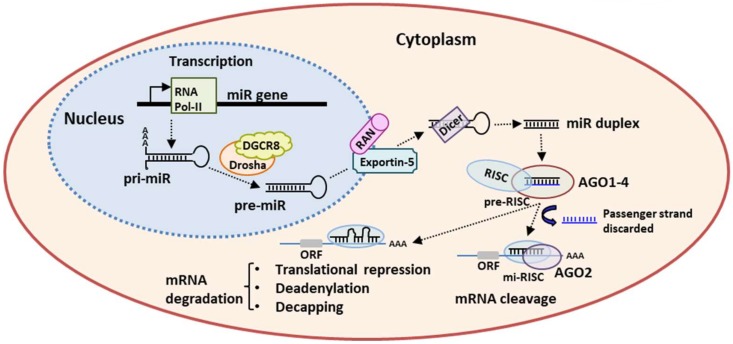
miRs processing and mechanism of action. RNA polymerase II (Pol-II) transcribes the primary miR transcript (pri-miR) subsequently cleaved by Drosha-DGCR8 complex into pre-miR. The resulting pre-miR is exported from the nucleus to the cytoplasm by Exportin-5/Ran-GTP. RNase Dicer cleaves the pre-miR to its mature miR duplex that is loaded onto Argonaute (AGO1–4) proteins and forms the pre-effector RNA-induced silencing complex (pre-RISC). The guide strand is retained into the mature miR-induced RISC (mi-RISC) whereas the passenger strand (blue) is discarded. A full complementary base pairing induces the mRNA cleavage by AGO2 slicing activity, while a partial complementary induces translational repression, deadenylation, and decapping followed by mRNA target degradation.

miRs are transcribed by RNA polymerase-II (Pol-II), and the transcription is controlled by epigenetic alterations, i.e., methylation and histone modification, and by several transcription factors-associated/non-associated to RNA Pol-II, including p53, MYC, and ZEB1/2 (Figure 1). 

RNA Pol-II generates the primary miR (pri-miR) longer than 1 kb, with a single-stranded RNA segment at 5′ and 3′ ends and a stem-loop structure that contains the sequence of mature miR [5]. Moreover, the nuclear RNA pol-III Drosha and its co-factor DiGeorge syndrome critical region 8 (DGCR8 or “Pasha”) form the microprocessor complex that cleaves pri-miR into pre-miR. The pre-miR is a hairpin RNA of 65 nucleotides that is actively exported from nucleus to cytoplasm by exportin 5/RAN·GTP [24]. Here, the pre-miR is processed by the RNAase III-type endonuclease Dicer that generates a small miR duplex of ≈22 nucleotides. The miR duplex is loaded onto an Agonauta (AGO) protein and forms the pre-effector complex, i.e., the RNA-induced silencing complex (pre-RISC). The AGO family consists of four AGO (AGO1–4) specialized small-RNA-binding proteins. In humans, AGO2 has a catalytic slicer activity that is able to cleave the phosphodiester bond of a target RNA and, thus, is involved in post-transcriptional gene silencing [25]. The pre-RISC releases one of the two strands of duplex miR, and generates the mature minimal miR-induced silencing complex (miRISC). Strand selection is based on the relative thermodynamic stability and on nucleotide sequence, in the form that the “guide strand” with lower 5′ stability and/or an uracil at 5′ ends is preferentially retained into miRISC, while the “passenger strand” is rapidly released and cleaved [5]. As the strand slicing is not a fine selective process, both the strands may be selected to generate the mature RISC complex resulting in two active miRs isoforms, the -5p and the -3p strands, arising from the 5′ end or the 3′ end of the pre-miR, respectively [5,26]. Finally, miRISC modulates gene expression by binding to a specific complementary sequence of mRNA target 3′ UTR to specific regions, i.e., the miRs response elements (MREs). The complementary miRs/MREs determines the mRNA fate: a full complementary base pairing induces the mRNA cleavage by AGO2 slicing activity [26]; a partial complementary base induces translational repression, deadenylation, and decapping, that is followed by mRNA target degradation [27,28]. For these reasons, one miR is able to target many mRNA targets implying that the deregulation of a single miR may affect several cellular processes [7].

## 3. miRs Deregulation in MM

The miRs are post-transcriptional regulators of gene expressions and modulate several biologic processes including cell differentiation, proliferation, apoptosis, survival, and metabolism that lead to cancer changeover [29,30]. Several studies documented miRs implication in the development of human solid and hematological tumors, including MM [29].

The miRs and mRNAs profiles were studied in preplasmablasts, plasmablasts, and in fully differentiated plasma cells (PCs) to identify the involvement of miRs in human PCs differentiation. The study pointed to three miRs clusters that finely regulate normal PCs differentiation and control their proliferation rate [31].

Epigenetic modifications, mutations, and defects of miRs biogenesis machinery may contribute to miRs downregulation and/or overexpression that may interfere with PCs differentiation and proliferation leading to malignant transformation [30].

The importance of miRs in the MM pathogenesis and progression has been highlighted by dissecting characteristic expression profiles. One of the underlying mechanisms leading to the evolution from monoclonal gammopathy of undetermined significance (MGUS) to MM is represented by distinctive expression patterns of different miRs between MGUS and MM PCs and normal PCs, also suggesting a role for miRs in MM progression. Pichiorri et al. [32] analyzed miRs expression in MM cell lines and in BM CD138^+^ cells purified from MM and MGUS patients and healthy donors. Microarray analysis revealed the overexpression of miR-21, miR-106b∼25 cluster, miR-181a/b in both MM and MGUS cells compared to normal PCs. Furthermore, they found higher levels of miR-32 and miR-17∼92 cluster in MM patients suggesting that the multistep progression of MM correlates to a gradual modulation of miRs expression.

Other studies confirmed the modulation of miRs profile during MM progression (Table 1). The recurrent altered miRs include miR-15a/miR-16-1 cluster, miR-21, miR-17∼92 cluster, and miR-34 family [12,15,32,33,34,35,36,37,38,39,40]. As listed in Table 1, they regulate the expression of gene/protein targets associated to cellular pathways that are deeply involved in MM pathogenesis, i.e., IL-6R/STAT-3, phosphoinositide 3-kinases (PI3K), mitogen-activated protein kinase (MAPK), p53, B-cell lymphoma-2 (Bcl-2), Cyclin D1, Notch, and vascular endothelial growth factor (VEGF). These pathways sustain cell proliferation, resistance to apoptosis and BM angiogenesis [12,15,32,33,34,35,36,37,38,39,40]. Furthermore, in vitro and in vivo studies support a tumor suppressor function of miR-29b. Its targets are the histone deacetylases (HDAC) that contribute to its downregulation via a feedback loop, and the DNA methyltransferase 3B (DNMT3B) [41,42]. The miR-29-b controls cell apoptosis, proliferation, and migration via epigenetic modifications and targeting the anti-apoptotic protein MCL-1 and the CDK6 [43]. Its levels are increased by bortezomib and enhance the bortezomib-induced apoptosis of MM cells via downregulation of the transcription factor Sp1 [44]. Data on the miR-125 family are controversial. The miR-125a-5p is overexpressed in MM patients and cell lines, and is closely associated to the t(4;14) translocation [45,46]. The miR-125a-5p reduces the expression of p53, p21, BAX, and MDM2, sustaining MM cell growth and migration and preventing cell apoptosis. By contrast, Morelli et al. [47] showed that miR-125b-5p is downregulated in CD138^+^ cells from MM patients and in MM cell lines suggesting its tumor suppressor role. Its enhanced expression abrogates the protective role of BMME, impairs MM cell growth and survival, and triggers apoptotic and autophagic-cell death. Conversely, a microarray analysis of circulating miRs showed that miR-125a-5p plasma levels have a diagnostic and predictive role in MM being correlated to disease stage and poor prognosis [48].

Overall, these studies established a direct link between miRs deregulation and malignant transformation suggesting their oncogenic and/or tumor suppressor role in MM (Table 1).

Recent studies documented that an aberrant miRs expression occurs not only in MM cells, but also in other BM cells [10,51,52,53,54,55,56,57]. Microarray analysis revealed that a miR deregulation occurs in BM fibroblasts (FBs) of MM versus MGUS suggesting that a specific aberrant miRs profile characterizes these cells in MM. The miR-27b-3p and miR-214-3p overexpression triggers cell proliferation and apoptosis resistance via the activation of FBXW7 and PTEN/AKT/GSK3 pathways driving the disease progression [51,52]. Furthermore, the miR-29b expression decreases during osteoclast differentiation contributing to MM-related bone disease [53], whereas its replacement restrains the bone resorption by MM cells [53]. Its replacement reduces RANK expression on the osteoclast cell membrane, thus reduces the production of pro-osteolytic enzymes, and restrains the bone resorption by MM cells [54]. The miR-29b downregulation is also involved in the creation of an immune suppressive BMME that contributes to disease progression. This downregulation was found in healthy dendritic cells (DCs) co-cultured with different MM cell lines as well as in CD11c^+^CD45^+^ DCs from MM patients versus normal mature DCs. In contrast, increased miR-29b expression counteracts the pro-inflammatory DCs phenotype preventing the intracellular pathways activation, i.e., nuclear factor-κB (NFkB), STAT3, mitogen-activated protein kinase and JUN. These signals sustain pro-inflammatory cytokine release and reduce the activation of pro-survival pathways in MM cells [54].

Analysis of extracellular vesicles (EVs) and/or exosomes demonstrated their contribution to miRs deregulation in BMME. Indeed, a microarray analysis of EVs released from BM stromal cells (SCs) identified a subset of miRs highly expressed in EVs but with low expression in BMSC_S_. The transfer of miR-10a to MM cells via EVs triggers cell proliferation in vitro and in vivo. By contrast, inhibition of the EVs release gives an increase of miR-10a expression in BMSCs that inhibits their proliferation and induces apoptosis. These data suggest that a selective miR transfer does occur into recipient cells and that the same miR may target different pathways in different cell types. This mechanism may contribute to create a tumor permissive microenvironment that sustains MM cell survival and growth [55]. Roccaro et al. [56] demonstrated that exosomal miRs cargo of BM mesenchymal SCs differs between MM and MGUS patients. MM BM mesenchymal SCs-derived exosomes have lower levels of the tumor suppressor miR-15a and higher levels of oncogenic proteins, cytokines, and adhesion molecules that sustain MM cells growth [56]. Furthermore, De Veirman et al. [57] showed that conditioned medium of MM cells modifies the miRs expression profile of mesenchymal SCs via exosome release. In particular, MM cells-derived exosomes induce the overexpression of miR-146a triggering the secretion of several cytokines/chemokines and sustaining MM cell viability and migration [57]. Finally, we demonstrated that MM cells-derived exosomes contain WWC-2 protein that activates Hippo signaling and induces de novo miRs synthesis in recipient FBs, suggesting the potential role of exosome in reprogramming BMME and miRs expression [51].

Overall, these studies support the idea that miRs deregulation is an important step of MM pathogenesis and progression and suggest that they may be envisaged as novel therapeutic management for MM patients.

## 4. Nanocarriers as miRs Delivery Systems

Nanocarriers as a miRs intracellular delivery system are considered a relevant strategy to improve the pharmacokinetic mechanisms in biological treatments thanks to their biodegradability and biocompatibility. Nanoparticles derived from lipids, polymers, and metals are used as a mechanism to transport miRs mimics and inhibitors [58]. Worth noting is that combination delivery of gene/drug using nanocarriers inhibits tumor growth and drug resistance far more than the treatment with genes or drugs alone, because the miRs mimics/inhibitors restore/ablate the miRs levels and may improve drug anti-tumor activity [59]. miRs mimics are double-stranded miRs-like RNA fragments with the 5′-end bearing a motif partially complementary to the selected sequence in the unique 3′UTR of the target gene [60]. Therefore, they act in a gene-specific way targeting specific mRNAs. Unlike by a native miR that operates on several genes a miRs mimic is able to specifically recognize only its target gene preventing the potential multiple side effects due to the inhibition of many target genes that may have different activity in different cell types [55,61]. miRs inhibitors are single stranded antisense oligonucleotides designed to bind with high-affinity and to inhibit endogenous-target mRNA [62,63].

Structurally, the phosphate backbone of miRs mimics/inhibitors has high hydrophilicity. Oligonucleotides phagocytosis and the subsequent engulfment into endosomes and lysosomes determine the degradation of miRs that do not reach their mRNA target. In addition, miRs mimics/inhibitors are eliminated from the blood circulation by nucleases, as well as by renal clearance due to their low molecular weight [64]. Accordingly, the new strategy to deliver miRs provides the use of biodegradable and biocompatible nanocarriers [58], i.e., lipid-based carriers, cationic polymer-based carriers, and exosomes to increase their stability and half-life (Table 2).

### 4.1. Lipid-Based Carriers

**Figure 2:**
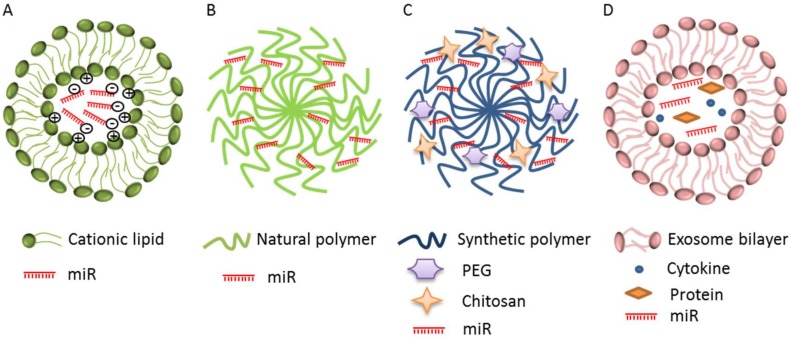
Schematic illustration of miRs delivery system. (**A**) Lipid-based carriers or cationic liposomes include monovalent and multivalent lipids; (**B**,**C**) polymer-based carriers include natural (green) and synthetic (blue) polymers conjugated with polyethylenglycol (PEG) and chitosan; (**D**) exosomes that vehicle miR, other nucleic acids (mRNA, lncRNA), cytokines, and proteins.

Lipid-based carriers (also now as cationic liposomes or lipoplexes) are a delivery system with a high transfection efficiency and biocompatibility [17,80]. Lipoplexes have a hydrophobic chain and a hydrophilic head group that spontaneously interact with the negative charge of the nucleic acid, and form stable lipoplexes via electrostatic interactions [16,81] (Figure 2A). Lipid-based carrier formation leads to both monovalent and multivalent aliphatic lipids. Monovalent lipids have a single functional amine in their head group, i.e., 1,2-di-O-octadecenyl-3-trimethylammonium propane (DOTMA), 1,2-dioleoyloxy-3-trimethylammonium propane (DOTAP), and dimethyldioctadecylammonium bromide (DDAB); while multivalent ones have many amine groups, i.e., 2,3-dioleyloxy-N-[2(sperminecarboxamido)ethyl]-N,N-dimethyl-l-propanaminium trifluoroacetate (DOSPA) and dioctadecylamidoglycylspermine (DOGS) [82]. Lipoplexes conjugated with cholesterol have high stability and can improve the membrane fusion increasing the transfection efficiency and the anti-tumor effects [83]. It is suggested that the inclusion of oleic acid (OA, unsaturated fatty acid) into the lipid-based nanoparticles significantly increases the miRs delivery [65].

Different lipid-based nanoparticles have been formulated. DOTMA have been investigated to target miR-133b and miR-29b for the treatment of lung cancer because of their inhibitory effect on cell proliferation and pro-apoptotic activity [66]. DOTMA-OA complexed with miR-122 was highly delivered in hepatocellular carcinoma (HCC) cells both in vitro and in vivo via intravenous injection. The cationic lipid DDAB complexed with the tumor suppressor miR-34a, inhibits tumor growth of lung metastasis in the murine B16F10-CD44^+^ melanoma model [69]. Abnormal miR-34a is highly expressed in MM, prostate cancer, kidney carcinoma, glioblastoma, and HCC [70,84,85,86]. It is downregulated in MM during osteoclast differentiation, thus it is a suppressor of osteoclastogenesis and bone resorption in the bone metastatic niche. It is targeted by transforming growth factor-b-induced factor 2 (Tgif2) [87]. Inhibition of Tgif2 reduces bone resorption and osteoporosis in bone metastases in vivo [87]. The miR-34a mimic encapsulated in SNALPs prompts an early inactivation of pro-survival and proliferative kinases extracellular signal-regulated kinases-2 (Erk-2) and AKT and an activation of caspase-6 and -3 followed by apoptosis induction [37]. The miR-34a/SNALPs efficiency inhibits the MM cell growth in vitro and reduces the growth of tumor xenografts in SCID mice in the absence of systemic toxicity [37,39].

### 4.2. Cationic Polymer-Based Carriers

Cationic polymer-based carriers are divided into natural and synthetic ones. Those natural are proteins, peptides, and polysaccharides, while synthetic ones involve dendrimers, polyethylenimines (PEIs), poly(lactic-co-glycolic acid) (PLGA), and polyphosphoesters [88] (Figure 2B,C). The above-mentioned delivery systems contain amine groups that interact with the phosphate groups of nucleic acids. Natural polymers include chitosan that is very effective due to its biocompatibility and biodegradability [89]. Although chitosan efficaciously binds and compacts nucleic acids, its delivery and transfection ability are often low in many cell lines. To avoid these problems its structure has been modified including thiolation, aminoethylation, and cholesterol [90].

PEIs are the most widely used cationic synthetic polymers. They are considered the gold standard of non-viral vectors due to their high transfection efficacy [91]. They are able to protect DNA from lysosomal degradation and cause lysosomal disruption as a result of the so-called proton sponge effect that enables DNA to be safely released into the cytoplasm [82]. PEIs have a strong charge density that allows the development of polyplexes with miRNAs, nevertheless, those compounds have high toxicity. To overcome this problem, PEIs have been modified by their conjugation with chitosan and polyethylene glycol (PEG) [91]. A PEI has been conjugated with miR-34a to inhibit proliferation and migration of prostate tumor cells [71]; with miR-145 and miR-33a to block growth of colon tumor cells [72]; and with miR-145 in vitro to promote apoptosis and reduce invasion of HCC cells [73].

PLGA is a hydrophobic polymer insoluble in water but soluble in organic solvents including dichloromethane, ethyl acetate, and chloroform; it is able to release prolonged the miRs. The capacity of PLGA nanoparticles to remain in the clathrin-coated vesicles for more than 72 h leads to slow internalization escaping lysosomal degradation [92]. The PLGA nanoparticles have been used to deliver miR-122, modulating apoptosis and inhibiting tumorigenesis in human colon tumor cells [74]. Babar et al. [75] employed PLGA to synthesize nanoparticles surface-coated with the cell-penetrating peptide penetratin for the delivery of antisense miR-155 to pre-B cell tumors, that decreased tumor growth in vivo.

The encapsulation of miR-34a into chitosan/PLGA nanoparticles leads to obtaining nanoplexes [76]. In vitro the miR34a-chitosan-PLGA inhibits the proliferation of RPMI-8226 and SKMM1 MM cell lines. The in vivo injection of miR-34a mimic-loaded nanoparticles significantly inhibits MM cell growth in NOD-SCID mice and improves their survival without organ toxicity [76].

### 4.3. Exosomes as miRs Delivery System

Lipid and polymeric nanoparticles are usually used as delivery systems for small molecules and anti-cancer drugs, but their ability to evade the immune system and their long circulating capability are still unknown. Exosomes are evaluated as a very good choice to overcome the limits of lipid and polymeric systems [93]. Exosomes are a kind of extracellular vesicles involved in the intercellular communication. They originate from endosomes and are secreted by different cell types into body fluids, i.e., blood, saliva, and urine [94]. They are nanospheres with a bilayer membrane that are able to transport miRs, other nucleic acids (i.e., mRNAs, lncRNAs), cytokines, and proteins that modulate the miRs expression in recipient cells, thus modifying the biological behavior of tumor and/or non-tumor cells [52,57,95] (Figure 2D).

The miRs delivery by exosomes is a strategy for the horizontal transfer of RNA [96]. The exosome transport acts as a model for cell-to-cell signaling by transferring miRs able to influence gene expression: this can affect cell phenotype and function and finally can promote the disease [97].

The miRs derived-exosomes participate in the crosstalk between MM cells and non-malignant components in an in vivo environment [77]. The role of circulating exosome-associated miRs in drug resistance was emphasized [77]. The downregulation of exosomal miR-16-5p, miR-15a-5p, miR-20a-5p, and miR-17-5p has been documented in the bortezomib resistant MM patients suggesting the identification of new markers for prediction of drug resistance and improving the understanding of in vivo intercellular crosstalk in these patients.

Let-7b and miR-18a in circulating exosomes can be considered markers for poor outcome in newly diagnosed MM patients [78]. Let-7 is significantly decreased in MM cells compared to normal PCs, and acts as a tumor-suppressor miR binding to deregulated LIN28B protein and inducing cell proliferation with the knocking down of CCND1, MYC, and RAS oncogenes [98,99,100]. The miR-18a is able to inhibit hypoxia-inducible factor-1α (HIF-1α) activity repressing tumor dissemination and mediating the activation of M1 macrophages by transcription factor interferon regulatory factor (IRF) 2. This process activates natural killer (NK) cells that play a critical role in the inhibition of tumor metastasis [101].

Since exosomes are considered as natural transporters of miRs and/or anti-miRs and paracrine mediators of cell-based therapy, they are used in several preclinical and clinical trials [102]. The BMSCs-derived exosomes are a delivery system for miR-146b [79]. This miR decreased epidermal growth factor receptor (EGFR) and NF-κB protein in 9L glioma cells in vitro, and inhibited small mothers against decapentaplegic (SMAD)-4, a protein of the SMAD family whose loss indicates poor outcome in glioma [103]. To test that BMSCs-derived exosomes can be used as a vehicle for delivery of anti-tumor miRs, BMSCs were transfected with miR-146b, and exosomes released by the BMSCs were harvested. Exosomes overexpressed miR-146b and were delivered via intra-tumor injection, reducing glioma xenograft growth in a rat model of primary brain tumor [79].

DCs-derived exosomes have been used in tumor immunotherapy [103,104,105,106], but among the different clinical applications no use in MM has been accurately identified yet.

### 4.4. miRs as Clinical-Based Therapeutic Strategies

Based on their active role in tumor progression and drug resistance, miRs are becoming promising therapeutic targets in clinical research for biotech companies and pharmaceutical industries. Phase I-II clinical trials designed for miRs target based therapy are ongoing [107].

In 2003, the miR-34 based-therapy MRX34 (Mirna Therapeutics, Inc.) was used to deliver a miR-34 mimic encapsulated in a liposomal nanoparticle formulation called NOV40 [108,109,110]. MRX-34 was identified as the first-in-class miR therapy for cancer. Patients with primary liver cancer, non-small cell lung cancer (NSCLC), lymphoma, melanoma, renal cell carcinoma (RCC), HCC, pancreatic cancer and MM were enrolled in phase I trials (ClinicalTrials.gov Id: NTC01829971) [108]. MRX34 was administered twice-weekly for 3 weeks in a 4-week cycle schedule and in the final part of the study, MRX34 was given intravenously daily for 5 days along with dexamethasone pre-medication twice daily for 7 days in week 1, followed by 2 weeks of rest in 3-week cycles [109]. Unfortunately, the trial was early closed due to serious immune-mediated adverse events that resulted in deaths of four patient with RCC, metastatic small cell lung cancer, metastatic melanoma and HCC, respectively. Data revealed no correlation between patients enrolled, adverse events and patients’ response in different cancer types and of particular note, no clinical data on MM have been discussed. Nevertheless, the study offers pharmacodynamics data to the proof-of-concept for miR-based cancer therapy. In particular, the clinical trial supports the dose-dependent modulation of miR-34a target genes in patients and the miR-34a importance in tumor development, suggesting the idea for pursuing miRNA mimic in cancer therapy. It remains unclear whether the MRX34 clinical toxicity and anti-tumor activity are related to specific gene-suppressing activity of the miR-34a nucleotide or to some other mechanisms [110]. Finally, authors suggested that improvement of new methods of miR delivery to the tumor able to avoid systemic immune activation was needed. 

Cortez et al. [111] demonstrated that miR-34 modulated p53 thus downregulating PDL1. They treated NSCLC patients with MRX34 in conjunction with radiotherapy, and obtained a reduction of PDL1 expression and of T-regulatory cells [111]. MiRagen started a multicenter phase-II clinical trial on patients with mycosis fungoides using a synthetic antagonist of miR-155 named MRG-106 (Cobomarsen, ClinicalTrials.gov Id: NCT03713320/phase II and NCT03837457/phase II) [63]. The miR-155 has a key role in the differentiation and proliferation of blood and lymphatic cells and its therapeutic inhibition restrains proliferation of lymphoma cells [112]. The MRG-106 treatment was evaluated as improvement of skin lesions, disease-associated symptoms, and quality of life. MRG-110 is a locked nucleic acid (LNA)-modified antisense oligonucleotide that inhibits miR-92 and is used to increase angiogenesis and the healing process in chronic ischemic disorders in an ongoing phase I clinical trial (ClinicalTrials.gov Id: NCT03603431/phase 1) [113]. RGLS5579 is an inhibitor of miR-10b. This miR is highly expressed in glioblastoma multiforme (GBM), and regulates cell proliferation, migration, and invasion [114]. The RGLS5579 is presently used in a clinical trial (Regulus Therapeutics Inc.) since preclinical studies have demonstrated its anti-tumor effect both in vitro and in vivo. A single dose of RGLS5579 as monotherapy increased survival in an orthotopic GBM animal model. RGLS5579 plus temozolomide also improved median survival with a good safety profile [115]. Finally, MesomiR-1 is a drug delivery vehicle (EDV, “nonliving bacterial nanoparticle”) complexed with a miR-16 mimic and a targeting moiety that is a bispecific antibody to EGFR and EDV. The EDVs are targeted to EGFR-expressing cancer cells by the anti-EGFR bispecific antibody. A phase I clinical trial on patients with malignant pleural mesothelioma and NSCLC is ongoing (ClinicalTrials.gov Id: NCT02369198) [116,117].

Overall, recent studies on nanocarriers improved miRs stability and delivery system supporting the potential use of miRs with co-delivery molecules in clinical management.

#### miRs as Clinical-Based Therapeutic Strategies in MM

As anti-MM therapies are based on combined strategies of multiple chemotherapeutic agents, the combination of miRs to anti-MM drugs regimens may improve the patients’ management and prevent drug resistance [118]. Preclinical studies investigated the potential synergistic effect of miRs inhibition/replacement with anti-MM drugs. The combination of the miR-34a with γ-secretase inhibitors enforces anti-MM related effect of miR-34a [119]. Zhao et al. [120] showed that inhibition of miR-221/222 reduced the drug resistance of MM1R MM cells to dexamethasone via upregulation of the pro-apoptotic PUMA in vitro and increased mice survival in vivo. Similarly, Gullà et al. [121] demonstrated that inhibition of miR-221/222 via LNA-i-miR-221 restores drug sensitivity in melphalan-refractory MM cells triggering cell apoptosis in vitro as well as in vivo. Furthermore, miR-29b replacement prevents the activation of the pro-survival autophagic pathway enhancing the anti-MM effect of bortezomib [122] and, finally, miR-21 inhibition in combination with other anti-MM drugs, i.e., dexamethasone, doxorubicin, and/or bortezomib, enhances MM cells sensitivity [123].

All these studies support the intriguing hypothesis of miRs targeting in combination with currently approved anti-MM drugs for the treatment of relapsed/refractory MM patients. However, the use of nanocarriers for miR delivery in combined regimens needs to be further investigated. To date, the only miRs-based clinical trial approved for the treatment of MM patients was the miR-34 based-therapy MRX34 as a single agent.

Finally, the theranostic potential use of miR has been investigated in MM to combine diagnostic with therapeutic strategies and to generate a personalized therapy that may improve patients’ outcome [124]. Studies uncovered primary samples-derived molecular fingerprints to be associated with MM progression and patients’ clinical outcome. This piece of evidence holds prognostic significance as well, since compelling data pinpoint a differential miR taxonomy in primary MM cells over the asymptomatic forms dyscrasias- and healthy donors-derived cells [124].

In conclusion, the role of miRs in the pathogenesis of cancer disease has been widely demonstrated. Their use as a therapeutic target still represents a challenge due to their cell-specific delivery, toxicity, and side effects. Although recent studies documented new strategies to ensure miRs cell-specific delivery by using nanoparticles coated with antibodies and/or ligands against oncogenic receptors expressed on the surface of cancer cells [125,126], their use in clinical practice needs to be further investigated. Overall, nanocarriers for miR delivery can be considered a great promise to introduce this innovative approach in nanomedicine for the treatment of cancer diseases including MM.

## Figures and Tables

**Table 1:** Deregulated miRs in multiple myeloma (MM).

miRNA	Target	Function in MM	Deregulation in MM	Reference
**miR-21**	PTEN	Proliferation and survival in vitro and in vivo	Overexpressed	[15,35]
	Rho-B			
	BTG2			
	AKT			
**miR-106b-25 cluster**	PCAF	Cell viability, colony formation	Overexpressed	[32,49]
	p38			
	MAPK			
**miR-181a/b**	BCL-2	Cell proliferation, apoptosis	Overexpressed	[32,50]
	NOVA1			
	PCAF			
**miR-15a/16-1 cluster**	Bcl-2	Proliferation, apoptosis, angiogenesis	Downregulated	[12,32,33]
	Cyclin D1			
	PI3K			
	MAPK			
	VEGF			
**miR-17-92 cluster**	SOCS-1	MM cells drug resistance, poor prognosis	Overexpressed	[32,36]
	BIM			
**miR-34 family**	c-MYC	Cell cycle, apoptosis, tumor growth in vivo	Downregulated	[39,40]
	CDK6			
	c-MET			
	Bcl-2			
	Notch1			
**miR-29b**	HDAC	Cell proliferation, apoptosis, migration	Downregulated	[41,42,43]
	DNMT3B			
	MCL-1			
	CDK-6			
	AKT			
	Sp1			
**miR-125a-5p**	p53	Cell growth, apoptosis, migration	Overexpressed	[44]
	p21			
	BAX			
	MDM2			
**miR-125b-5p**	IRF4	Cell growth, apoptosis, autophagy	Downregulated	[47]

**Table 2:** Nanocarriers used as therapeutic delivery systems of miRs.

Carrier Type	Delivery System	Targeted miRNA	Cancer Type	Reference
** Lipid-based Carriers **	DOTMA	miR-122	Liver cancer Lung cancer	[65]
		miR-133b		[66]
		miR-29b		[67]
	DOTAP	let-7a miR	Lung cancer Mesothelioma	[68]
	DDAB	miR-34a	Lung cancer Melanoma	[69]
	SNALPs	miR-34a	Multiple Myeloma	[70]
** Cationic Polymer-Based Carriers **	PEI	miR-34a	Prostate cancer	[71]
		miR-145	HCC	[72]
		miR-33a	Colon cancer	[73]
	PLGA	miR-122	Colon cancer	[74]
		miR-155	Lymphoma	[75]
	Chitosan/PLGA	miR-34a	Multiple Myeloma	[76]
**Exososome**		miR-16-5p	Multiple Myeloma	[77]
		miR-15a-5p		
		miR-20a-5p		
		miR17-5p		
		let-7b	Multiple Myeloma	[78]
		miR-18a		
		miR-27b-3p	Multiple Myeloma	[52]
		miR-214-3p		
		miR-146b	Glioma	[79]

DOTMA = 1,2-di-O-octadecenyl-3-trimethylammonium propane; DOTAP = 1,2-dioleoyloxy-3-trimethylammonium propane; dimethyldioctadecylammonium bromide; SNALP = stable nucleic acid lipid particle; PEI = polyethylenimine; PLGA = poly(lactic-co-glycolic acid).

## References

[1] Anderson K.C., Carrasco R.D. (2011). Pathogenesis of myeloma. Annu. Rev. Pathol..

[2] Di Marzo L., Desantis V., Solimando A.G., Ruggieri S., Annese T., Nico B., Fumarulo R., Vacca A., Frassanito M.A. (2016). Microenvironment drug resistance in multiple myeloma: Emerging new players. Oncotarget.

[3] Solimando A.G., Da Vià M.C., Cicco S., Leone P., Di Lernia G., Giannico D., Desantis V., Frassanito M.A., Morizio A., Delgado Tascon J. (2019). High-Risk Multiple Myeloma: Integrated Clinical and Omics Approach Dissects the Neoplastic Clone and the Tumor Microenvironment. J. Clin. Med..

[4] He L., Hannon G.J. (2004). MicroRNAs: Small RNAs with a big role in gene regulation. Nat. Rev. Genet..

[5] Ha M., Kim V.N. (2014). Regulation of microRNA biogenesis. Nat. Rev. Mol. Cell Biol..

[6] Iorio M.V., Croce C.M. (2012). MicroRNA dysregulation in cancer: Diagnostics, monitoring and therapeutics. EMBO Mol. Med..

[7] Bartel D.P. (2004). MicroRNAs: Genomics, biogenesis, mechanism, and function. Cell.

[8] Chitkara D., Mittal A., Mahato R.I. (2015). miRNAs in pancreatic cancer: Therapeutic potential, delivery challenges and strategies. Adv. Drug Deliv. Rev..

[9] Benetatos L., Vartholomatos G. (2012). Deregulated microRNAs in multiple myeloma. Cancer.

[10] Tagliaferri P., Rossi M., Di Martino M.T., Amodio N., Leone E., Gulla A., Neri A., Tassone P. (2012). Promises and challenges of MicroRNA-based treatment of multiple myeloma. Curr. Cancer Drug Targets.

[11] Handa H., Murakami Y., Ishihara R., Kimura-Masuda K., Masuda Y. (2019). The Role and Function of microRNA in the Pathogenesis of Multiple Myeloma. Cancers.

[12] Roccaro A.M., Sacco A., Thompson B., Leleu X., Azab A.K., Azab F., Runnels J., Jia X., Ngo H.T., Melhem M.R. (2009). Micro RNAs 15a and 16 regulate tumor proliferation in multiple myeloma. Blood.

[13] Svoronos A.A., Engelman D.M., Slack F.J. (2016). OncomiR or Tumor Suppressor? The Duplicity of MicroRNAs in Cancer. Cancer Res..

[14] Zhang Y., Wang Z., Gemeinhart R.A. (2013). Progress in microRNA delivery. J. Control. Release.

[15] Munker R., Liu C.G., Taccioli C., Alder H., Heerema N. (2010). MicroRNA profiles of drug-resistant myeloma cell lines. Acta Haematol..

[16] Chitkara D., Singh S., Mittal A. (2016). Nanocarrier-based co-delivery of small molecules and siRNA/miRNA for treatment of cancer. Ther. Deliv..

[17] Scheideler M., Vidakovic I., Prassl R. (2020). Lipid nanocarriers for microRNA delivery. Chem. Phys. Lipids.

[18] Shi Z., Chen Q., Li C., Wang L., Qian X., Jiang C., Liu X., Wang X., Li H., Kang C. (2014). MiR-124 governs glioma growth and angiogenesis and enhances chemosensitivity by targeting R-Ras and N-Ras. Neuro Oncol..

[19] Qian X., Ren Y., Shi Z., Long L., Pu P., Shen J., Yuan X., Kang C. (2012). Sequence-dependent synergistic inhibition of human glioma cell lines by combined temozolomide and miR-21 inhibitor gene therapy. Mol. Pharm..

[20] Gandhi N.S., Tekade R.K., Chougule M.B. (2014). Nanocarrier mediated delivery of siRNA/miRNA in combination with chemotherapeutic agents for cancer therapy: Current progress and advances. J. Control. Release.

[21] Lee Y., Jeon K., Lee J.T., Kim S., Kim V.N. (2002). MicroRNA maturation: Stepwise processing and subcellular localization. EMBO J..

[22] Singh A.K., Singh N., Kumar S., Kumari J., Singh R., Gaba S., Yadav M.C., Grover M., Chaurasia S., Kumar R. (2020). Identification and evolutionary analysis of polycistronic miRNA clusters in domesticated and wild wheat. Genomics.

[23] Monteys A.M., Spengler R.M., Wan J., Tecedor L., Lennox K.A., Xing Y., Davidson B.L. (2010). Structure and activity of putative intronic miRNA promoters. RNA.

[24] Krol J., Loedige I., Filipowicz W. (2010). The widespread regulation of microRNA biogenesis, function and decay. Nat. Rev. Genet..

[25] Niaz S. (2018). The AGO proteins: An overview. Biol. Chem..

[26] O’Brien J., Hayder H., Zayed Y., Peng C. (2018). Overview of MicroRNA Biogenesis, Mechanisms of Actions, and Circulation. Front. Endocrinol. (Lausanne).

[27] Fabian M.R., Sonenberg N. (2012). The mechanics of miRNA-mediated gene silencing: A look under the hood of miRISC. Nat. Struct. Mol. Biol..

[28] Wilczynska A., Bushell M. (2015). The complexity of miRNA-mediated repression. Cell Death Differ..

[29] Croce C.M. (2009). Causes and consequences of microRNA dysregulation in cancer. Nat. Rev. Genet..

[30] Lionetti M., Agnelli L., Lombardi L., Tassone P., Neri A. (2012). MicroRNAs in the pathobiology of multiple myeloma. Curr. Cancer Drug Targets.

[31] Kassambara A., Jourdan M., Bruyer A., Robert N., Pantesco V., Elemento O., Klein B., Moreaux J. (2017). Global miRNA expression analysis identifies novel key regulators of plasma cell differentiation and malignant plasma cell. Nucleic Acids Res..

[32] Pichiorri F., Suh S.S., Ladetto M., Kuehl M., Palumbo T., Drandi D., Taccioli C., Zanesi N., Alder H., Hagan J.P. (2008). MicroRNAs regulate critical genes associated with multiple myeloma pathogenesis. Proc. Natl. Acad. Sci. USA.

[33] Sun C.Y., She X.M., Qin Y., Chu Z.B., Chen L., Ai L.S., Zhang L., Hu Y. (2013). miR-15a and miR-16 affect the angiogenesis of multiple myeloma by targeting VEGF. Carcinogenesis.

[34] Loffler D., Brocke-Heidrich K., Pfeifer G., Stocsits C., Hackermuller J., Kretzschmar A.K., Burger R., Gramatzki M., Blumert C., Bauer K. (2007). Interleukin-6 dependent survival of multiple myeloma cells involves the Stat3-mediated induction of microRNA-21 through a highly conserved enhancer. Blood.

[35] Leone E., Morelli E., Di Martino M.T., Amodio N., Foresta U., Gullà A., Rossi M., Neri A., Giordano A., Munshi N.C. (2013). Targeting miR-21 inhibits in vitro and in vivo multiple myeloma cell growth. Clin. Cancer Res..

[36] Petrocca F., Visone R., Onelli M.R., Shah M.H., Nicoloso M.S., de Martino I., Iliopoulos D., Pilozzi E., Liu C.G., Negrini M. (2008). E2F1-regulated microRNAs impair TGFbeta-dependent cell-cycle arrest and apoptosis in gastric cancer. Cancer Cell.

[37] Chen L., Li C., Zhang R., Gao X., Qu X., Zhao M., Qiao C., Xu J., Li J. (2011). miR-17-92 cluster microRNAs confers tumorigenicity in multiple myeloma. Cancer Lett..

[38] Chim C.S., Wong K.Y., Qi Y., Loong F., Lam W.L., Wong L.G., Jin D.Y., Costello J.F., Liang R. (2010). Epigenetic inactivation of the miR-34a in hematological malignancies. Carcinogenesis.

[39] Misso G., Di Martino M.T., De Rosa G., Farooqi A.A., Lombardi A., Campani V., Zarone M.R., Gullà A., Tagliaferri P., Tassone P. (2014). Mir-34: A new weapon against cancer?. Mol. Ther. Nucleic Acids.

[40] Di Martino M.T., Campani V., Misso G., Gallo Cantafio M.E., Gullà A., Foresta U., Guzzi P.H., Castellano M., Grimaldi A., Gigantino V. (2014). In vivo activity of miR-34a mimics delivered by stable nucleic acid lipid particles (SNALPs) against multiple myeloma. PLoS ONE.

[41] Amodio N., Stamato M.A., Gullà A.M., Morelli E., Romeo E., Raimondi L., Pitari M.R., Ferrandino I., Misso G., Caraglia M. (2016). Therapeutic Targeting of miR-29b/HDAC4 Epigenetic Loop in Multiple Myeloma. Mol. Cancer Ther..

[42] Amodio N., Leotta M., Bellizzi D., Di Martino M.T., D’Aquila P., Lionetti M., Fabiani F., Leone E., Gullà A.M., Passarino G. (2012). DNA-demethylating and anti-tumor activity of synthetic miR-29b mimics in multiple myeloma. Oncotarget.

[43] Zhang Y.K., Wang H., Leng Y., Li Z.L., Yang Y.F., Xiao F.J., Li Q.F., Chen X.Q., Wang L.S. (2011). Overexpression of microRNA-29b induces apoptosis of multiple myeloma cells through down regulating Mcl-1. Biochem. Biophys. Res. Commun..

[44] Amodio N., Di Martino M.T., Foresta U., Leone E., Lionetti M., Leotta M., Gullà A.M., Pitari M.R., Conforti F., Rossi M. (2012). miR-29b sensitizes multiple myeloma cells to bortezomib-induced apoptosis through the activation of a feedback loop with the transcription factor Sp1. Cell Death Dis..

[45] Lionetti M., Biasiolo M., Agnelli L., Todoerti K., Mosca L., Fabris S., Sales G., Deliliers G.L., Bicciato S., Lombardi L. (2009). Identification of microRNA expression patterns and definition of a microRNA/mRNA regulatory network in distinct molecular groups of multiple myeloma. Blood.

[46] Leotta M., Biamonte L., Raimondi L., Ronchetti D., Di Martino M.T., Botta C., Leone E., Pitari M.R., Neri A., Giordano A. (2014). A p53-dependent tumor suppressor network is induced by selective miR-125a-5p inhibition in multiple myeloma cells. J. Cell Physiol..

[47] Morelli E., Leone E., Cantafio M.E., Di Martino M.T., Amodio N., Biamonte L., Gullà A., Foresta U., Pitari M.R., Botta C. (2015). Selective targeting of IRF4 by synthetic microRNA-125b-5p mimics induces anti-multiple myeloma activity in vitro and in vivo. Leukemia.

[48] Jiang Y., Luan Y., Chang H., Chen G. (2018). The diagnostic and prognostic value of plasma microRNA-125b-5p in patients with multiple myeloma. Oncol. Lett..

[49] Gu C., Li T., Yin Z., Chen S., Fei J., Shen J., Zhang Y. (2017). Integrative analysis of signaling pathways and diseases associated with the miR-106b/25 cluster and their function study in berberine-induced multiple myeloma cells. Funct. Integr. Genom..

[50] Yuan R., Liu N., Yang J., Peng J., Liu L., Guo X. (2018). The expression and role of miR-181a in multiple myeloma. Medicine (Baltimore).

[51] Frassanito M.A., Desantis V., Di Marzo L., Craparotta I., Beltrame L., Marchini S., Annese T., Visino F., Arciuli M., Saltarella I. (2019). Bone marrow fibroblasts overexpress miR-27b and miR-214 in step with multiple myeloma progression, dependent on tumour cell-derived exosomes. J. Pathol..

[52] Mori M., Triboulet R., Mohseni M., Schlegelmilch K., Shrestha K., Camargo F.D., Gregory R.I. (2014). Hippo signaling regulates microprocessor and links cell-density-dependent miRNA biogenesis to cancer. Cell.

[53] Rossi M., Pitari M.R., Amodio N., Di Martino M.T., Conforti F., Leone E., Botta C., Paolino F.M., Del Giudice T., Iuliano E. (2013). miR-29b negatively regulates human osteoclastic cell differentiation and function: Implications for the treatment of multiple myeloma-related bone disease. J. Cell Physiol..

[54] Botta C., Cucè M., Pitari M.R., Caracciolo D., Gullà A., Morelli E., Riillo C., Biamonte L., Gallo Cantafio M.E., Prabhala R. (2018). MiR-29b antagonizes the pro-inflammatory tumor-promoting activity of multiple myeloma-educated dendritic cells. Leukemia.

[55] Umezu T., Imanishi S., Yoshizawa S., Kawana C., Ohyashiki J.H., Ohyashiki K. (2019). Induction of multiple myeloma bone marrow stromal cell apoptosis by inhibiting extracellular vesicle miR-10a secretion. Blood Adv..

[56] Roccaro A.M., Sacco A., Maiso P., Azab A.K., Tai Y.T., Reagan M., Azab F., Flores L.M., Campigotto F., Weller E. (2013). BM mesenchymal stromal cell-derived exosomes facilitate multiple myeloma progression. J. Clin. Investig..

[57] De Veirman K., Wang J., Xu S., Leleu X., Himpe E., Maes K., De Bruyne E., Van Valckenborgh E., Vanderkerken K., Menu E. (2016). Induction of miR-146a by multiple myeloma cells in mesenchymal stromal cells stimulates their pro-tumoral activity. Cancer Lett..

[58] Chen Y., Gao D.Y., Huang L. (2015). In vivo delivery of miRNAs for cancer therapy: Challenges and strategies. Adv. Drug Deliv. Rev..

[59] Kang L., Gao Z., Huang W., Jin M., Wang Q. (2015). Nanocarrier-mediated co-delivery of chemotherapeutic drugs and gene agents for cancer treatment. Acta Pharm. Sin. B.

[60] Wang Z. (2011). The guideline of the design and validation of MiRNA mimics. Methods Mol. Biol..

[61] Hosseinahli N., Aghapour M., Duijf P.H.G., Baradaran B. (2018). Treating cancer with microRNA replacement therapy: A literature review. J. Cell Physiol..

[62] Lima J.F., Cerqueira L., Figueiredo C., Oliveira C., Azevedo N.F. (2018). Anti-miRNA oligonucleotides: A comprehensive guide for design. RNA Biol..

[63] Rupaimoole R., Slack F.J. (2017). MicroRNA therapeutics: Towards a new era for the management of cancer and other diseases. Nat. Rev. Drug Discov..

[64] Bader A.G., Brown D., Stoudemire J., Lammers P. (2011). Developing therapeutic microRNAs for cancer. Gene Ther..

[65] Wang X., Yu B., Ren W., Mo X., Zhou C., He H., Jia H., Wang L., Jacob S.T., Lee R.J. (2013). Enhanced hepatic delivery of siRNA and microRNA using oleic acid based lipid nanoparticle formulations. J. Control. Release.

[66] Wu Y., Crawford M., Yu B., Mao Y., Nana-Sinkam S.P., Lee L.J. (2011). MicroRNA delivery by cationic lipoplexes for lung cancer therapy. Mol. Pharm..

[67] Wu Y., Crawford M., Mao Y., Lee R.J., Davis I.C., Elton T.S., Lee L.J., Nana-Sinkam S.P. (2013). Therapeutic Delivery of MicroRNA-29b by Cationic Lipoplexes for Lung Cancer. Mol. Ther. Nucleic Acids.

[68] Lee H.Y., Mohammed K.A., Kaye F., Sharma P., Moudgil B.M., Clapp W.L., Nasreen N. (2013). Targeted delivery of let-7a microRNA encapsulated ephrin-A1 conjugated liposomal nanoparticles inhibit tumor growth in lung cancer. Int. J. Nanomed..

[69] Shi S., Han L., Deng L., Zhang Y., Shen H., Gong T., Zhang Z., Sun X. (2014). Dual drugs (microRNA-34a and paclitaxel)-loaded functional solid lipid nanoparticles for synergistic cancer cell suppression. J. Control. Release.

[70] He L., He X., Lim L.P., de Stanchina E., Xuan Z., Liang Y., Xue W., Zender L., Magnus J., Ridzon D. (2007). A microRNA component of the p53 tumour suppressor network. Nature.

[71] Jung H., Kim S.A., Yang Y.G., Yoo H., Lim S.J., Mok H. (2015). Long chain microRNA conjugates in calcium phosphate nanoparticles for efficient formulation and delivery. Arch. Pharm. Res..

[72] Ibrahim A.F., Weirauch U., Thomas M., Grünweller A., Hartmann R.K., Aigner A. (2011). MicroRNA replacement therapy for miR-145 and miR-33a is efficacious in a model of colon carcinoma. Cancer Res..

[73] Che H.L., Lee H.J., Uto K., Ebara M., Kim W.J., Aoyagi T., Park I.K. (2015). Simultaneous Drug and Gene Delivery from the Biodegradable Poly(ε-caprolactone) Nanofibers for the Treatment of Liver Cancer. J. Nanosci. Nanotechnol..

[74] Wang T.Y., Choe J.W., Pu K., Devulapally R., Bachawal S., Machtaler S., Chowdhury S.M., Luong R., Tian L., Khuri-Yakub B. (2015). Ultrasound-guided delivery of microRNA loaded nanoparticles into cancer. J. Control. Release.

[75] Babar I.A., Cheng C.J., Booth C.J., Liang X., Weidhaas J.B., Saltzman W.M., Slack F.J. (2012). Nanoparticle-based therapy in an in vivo microRNA-155 (miR-155)-dependent mouse model of lymphoma. Proc. Natl. Acad. Sci. USA.

[76] Cosco D., Cilurzo F., Maiuolo J., Federico C., Di Martino M.T., Cristiano M.C., Tassone P., Fresta M., Paolino D. (2015). Delivery of miR-34a by chitosan/PLGA nanoplexes for the anticancer treatment of multiple myeloma. Sci. Rep..

[77] Zhang L., Pan L., Xiang B., Zhu H., Wu Y., Chen M., Guan P., Zou X., Valencia C.A., Dong B. (2016). Potential role of exosome-associated microRNA panels and in vivo environment to predict drug resistance for patients with multiple myeloma. Oncotarget.

[78] Manier S., Liu C.J., Avet-Loiseau H., Park J., Shi J., Campigotto F., Salem K.Z., Huynh D., Glavey S.V., Rivotto B. (2017). Prognostic role of circulating exosomal miRNAs in multiple myeloma. Blood.

[79] Katakowski M., Buller B., Zheng X., Lu Y., Rogers T., Osobamiro O., Shu W., Jiang F., Chopp M. (2013). Exosomes from marrow stromal cells expressing miR-146b inhibit glioma growth. Cancer Lett..

[80] Xia Y., Tian J., Chen X. (2016). Effect of surface properties on liposomal siRNA delivery. Biomaterials.

[81] Anwer K., Meaney C., Kao G., Hussain N., Shelvin R., Earls R.M., Leonard P., Quezada A., Rolland A.P., Sullivan S.M. (2000). Cationic lipid-based delivery system for systemic cancer gene therapy. Cancer Gene Ther..

[82] Muthiah M., Park I.K., Cho C.S. (2013). Nanoparticle-mediated delivery of therapeutic genes: Focus on miRNA therapeutics. Expert Opin. Drug Deliv..

[83] Anderson D.M., Hall L.L., Ayyalapu A.R., Irion V.R., Nantz M.H., Hecker J.G. (2003). Stability of mRNA/cationic lipid lipoplexes in human and rat cerebrospinal fluid: Methods and evidence for nonviral mRNA gene delivery to the central nervous system. Hum. Gene Ther..

[84] Welch C., Chen Y., Stallings R.L. (2007). MicroRNA-34a functions as a potential tumor suppressor by inducing apoptosis in neuroblastoma cells. Oncogene.

[85] Liu C., Kelnar K., Liu B., Chen X., Calhoun-Davis T., Li H., Patrawala L., Yan H., Jeter C., Honorio S. (2011). The microRNA miR-34a inhibits prostate cancer stem cells and metastasis by directly repressing CD44. Nat. Med..

[86] Wang Y., Wang C.M., Jiang Z.Z., Yu X.J., Fan C.G., Xu F.F., Zhang Q., Li L.I., Li R.F., Sun W.S. (2015). MicroRNA-34c targets TGFB-induced factor homeobox 2, represses cell proliferation and induces apoptosis in hepatitis B virus-related hepatocellular carcinoma. Oncol. Lett..

[87] Krzeszinski J.Y., Wei W., Huynh H., Jin Z., Wang X., Chang T.C., Xie X.J., He L., Mangala L.S., Lopez-Berestein G. (2014). miR-34a blocks osteoporosis and bone metastasis by inhibiting osteoclastogenesis and Tgif2. Nature.

[88] Srinivasachari S., Zhang G.D. (2004). Novel cationic polymers and glycodendrimers for gene delivery. Pap. Am. Chem..

[89] Erbacher P., Zou S., Bettinger T., Steffan A.M., Remy J.S. (1998). Chitosan-based vector/DNA complexes for gene delivery: Biophysical characteristics and transfection ability. Pharm. Res..

[90] Zhang Y., Chen J., Zhang Y., Pan Y., Zhao J., Ren L., Liao M., Hu Z., Kong L., Wang J. (2007). A novel PEGylation of chitosan nanoparticles for gene delivery. Biotechnol. Appl. Biochem..

[91] Fernandez-Piñeiro I., Badiola I., Sanchez A. (2017). Nanocarriers for microRNA delivery in cancer medicine. Biotechnol. Adv..

[92] Devalliere J., Chang W.G., Andrejecsk J.W., Abrahimi P., Cheng C.J., Jane-wit D., Saltzman W.M., Pober J.S. (2014). Sustained delivery of proangiogenic microRNA-132 by nanoparticle transfection improves endothelial cell transplantation. FASEB J..

[93] Turturici G., Tinnirello R., Sconzo G., Geraci F. (2014). Extracellular membrane vesicles as a mechanism of cell-to-cell communication: Advantages and disadvantages. Am. J. Physiol. Cell Physiol..

[94] Daßler-Plenker J., Küttner V., Egeblad M. (2020). Communication in tiny packages: Exosomes as means of tumor-stroma communication. Biochim. Biophys. Acta Rev. Cancer.

[95] Whiteside T.L., Boyiadzis M. (2017). Response commentary: Exosomes vs microvesicles in hematological malignancies. Leukemia.

[96] Ramachandran S., Palanisamy V. (2012). Horizontal transfer of RNAs: Exosomes as mediators of intercellular communication. Wiley Interdiscip. Rev. RNA.

[97] Squadrito M.L., Baer C., Burdet F., Maderna C., Gilfillan G.D., Lyle R., Ibberson M., De Palma M. (2014). Endogenous RNAs modulate microRNA sorting to exosomes and transfer to acceptor cells. Cell Rep..

[98] Manier S., Powers J.T., Sacco A., Glavey S.V., Huynh D., Reagan M.R., Salem K.Z., Moschetta M., Shi J., Mishima Y. (2017). The LIN28B/let-7 axis is a novel therapeutic pathway in multiple myeloma. Leukemia.

[99] Spizzo R., Nicoloso M.S., Croce C.M., Calin G.A. (2009). SnapShot: MicroRNAs in Cancer. Cell.

[100] Büssing I., Slack F.J., Grosshans H. (2008). let-7 microRNAs in development, stem cells and cancer. Trends Mol. Med..

[101] Krutilina R., Sun W., Sethuraman A., Brown M., Seagroves T.N., Pfeffer L.M., Ignatova T., Fan M. (2014). MicroRNA-18a inhibits hypoxia-inducible factor 1α activity and lung metastasis in basal breast cancers. Breast Cancer Res..

[102] Di Rocco G., Baldari S., Toietta G. (2017). Exosomes and other extracellular vesicles-mediated microRNA delivery for cancer therapy. Transl. Cancer Res..

[103] Geraldo M.V., Yamashita A.S., Kimura E.T. (2012). MicroRNA miR-146b-5p regulates signal transduction of TGF-β by repressing SMAD4 in thyroid cancer. Oncogene.

[104] Escudier B., Dorval T., Chaput N., André F., Caby M.P., Novault S., Flament C., Leboulaire C., Borg C., Amigorena S. (2005). Vaccination of metastatic melanoma patients with autologous dendritic cell (DC) derived-exosomes: Results of thefirst phase I clinical trial. J. Transl. Med..

[105] Dai S., Wei D., Wu Z., Zhou X., Wei X., Huang H., Li G. (2008). Phase I clinical trial of autologous ascites-derived exosomes combined with GM-CSF for colorectal cancer. Mol. Ther..

[106] Morse M.A., Garst J., Osada T., Khan S., Hobeika A., Clay T.M., Valente N., Shreeniwas R., Sutton M.A., Delcayre A. (2005). A phase I study of dexosome immunotherapy in patients with advanced non-small cell lung cancer. J. Transl. Med..

[107] Hanna J., Hossain G.S., Kocerha J. (2019). The Potential for microRNA Therapeutics and Clinical Research. Front. Genet..

[108] Hong D.S., Yoon-Koo K., Brenner A.J., Sachdev J.C., Ejadi S., Borad M.J., Kim T.Y., Lim H.Y., Park K., Becerra C. (2016). MRX34, a liposomal miR-34 mimic, in patients with advanced solid tumors: Final dose-escalation results from a first-in-human phase I trial of microRNA therapy. J. Clin. Oncol..

[109] Beg M.S., Brenner A.J., Sachdev J., Borad M., Kang Y.K., Stoudemire J., Smith S., Bader A.G., Kim S., Hong D.S. (2017). Phase I study of MRX34, a liposomal miR-34a mimic, administered twice weekly in patients with advanced solid tumors. Invest New Drugs.

[110] Hong D.S., Kang Y.K., Borad M., Sachdev J., Ejadi S., Lim H.Y., Brenner A.J., Park K., Lee J.L., Kim T.Y. (2020). Phase 1 study of MRX34, a liposomal miR-34a mimic, in patients with advanced solid tumours. Br. J. Cancer.

[111] Cortez M.A., Ivan C., Valdecanas D., Wang X., Peltier H.J., Ye Y., Araujo L., Carbone D.P., Shilo K., Giri D.K. (2015). PDL1 Regulation by p53 via miR-34. J. Natl. Cancer Inst..

[112] Li X.D., Li X.M., Gu J.W., Sun X.C. (2017). MiR-155 regulates lymphoma cell proliferation and apoptosis through targeting SOCS3/JAK-STAT3 signaling pathway. Eur. Rev. Med. Pharmacol. Sci..

[113] Deng S., Zhang Y., Wang Y., Lu X., Jiang Q. (2019). MicroRNA-92 regulates vascular smooth muscle cell function by targeting KLF4 during vascular restenosis and injury. Int. J. Clin. Exp. Pathol..

[114] Zhen L., Li J., Zhang M., Yang K. (2016). MiR-10b decreases sensitivity of glioblastoma cells to radiation by targeting AKT. J. Biol. Res..

[115] Ghosh D., Nandi S., Bhattacharjee S. (2018). Combination therapy to checkmate Glioblastoma: Clinical challenges and advances. Clin. Transl. Med..

[116] Van Zandwijk N., McDiarmid J., Brahmbhatt H., Reid G. (2018). Response to “An innovative mesothelioma treatment based on mir-16 mimic loaded EGFR targeted minicells (TargomiRs)”. Transl. Lung Cancer Res..

[117] Reid G., Kao S.C., Pavlakis N., Brahmbhatt H., MacDiarmid J., Clarke S., Boyer M., van Zandwijk N. (2016). Clinical development of TargomiRs, a miRNA mimic-based treatment for patients with recurrent thoracic cancer. Epigenomics.

[118] Zhu B., Ju S., Chu H., Shen X., Zhang Y., Luo X., Cong H. (2018). The potential function of microRNAs as biomarkers and therapeutic targets in multiple myeloma. Oncol. Lett..

[119] Zarone M.R., Misso G., Grimaldi A., Zappavigna S., Russo M., Amler E., Di Martino M.T., Amodio N., Tagliaferri P., Tassone P. (2017). Evidence of novel miR-34a-based therapeutic approaches for multiple myeloma treatment. Sci. Rep..

[120] Zhao J.J., Chu Z.B., Hu Y., Lin J., Wang Z., Jiang M., Chen M., Wang X., Kang Y., Zhou Y. (2015). Targeting the miR-221-222/PUMA/BAK/BAX pathway abrogates dexamethasone resistance in multiple myeloma. Cancer Res..

[121] Gullà A., Di Martino M.T., Gallo Cantafio M.E., Morelli E., Amodio N., Botta C., Pitari M.R., Lio S.G., Britti D., Stamato M.A. (2016). A 13 mer LNA-i-miR-221 Inhibitor Restores Drug Sensitivity in Melphalan-Refractory Multiple Myeloma Cells. Clin. Cancer Res..

[122] Jagannathan S., Vad N., Vallabhapurapu S., Vallabhapurapu S., Anderson K.C., Driscoll J.J. (2015). MiR-29b replacement inhibits proteasomes and disrupts aggresome+autophagosome formation to enhance the antimyeloma benefit of bortezomib. Leukemia.

[123] Wang X., Li C., Ju S., Wang Y., Wang H., Zhong R. (2011). Myeloma cell adhesion to bone marrow stromal cells confers drug resistance by microRNA-21 up-regulation. Leuk. Lymphoma.

[124] Ahmad N., Haider S., Jagannathan S., Anaissie E., Driscoll J.J. (2014). MicroRNA theragnostics for the clinical management of multiple myeloma. Leukemia.

[125] Esposito C.L., Cerchia L., Catuogno S., De Vita G., Dassie J.P., Santamaria G., Swiderski P., Condorelli G., Giangrande P.H., de Franciscis V. (2014). Multifunctional aptamer-miRNA conjugates for targeted cancer therapy. Mol Ther..

[126] Ganju A., Khan S., Hafeez B.B., Behrman S.W., Yallapu M.M., Chauhan S.C., Jaggi M. (2017). miRNA nanotherapeutics for cancer. Drug Discov. Today.

